# Role of the Extracellular and Intracellular Loops of Follicle-Stimulating Hormone Receptor in Its Function

**DOI:** 10.3389/fendo.2015.00110

**Published:** 2015-07-17

**Authors:** Antara A. Banerjee, Smita D. Mahale

**Affiliations:** ^1^Division of Structural Biology, National Institute for Research in Reproductive Health, Indian Council of Medical Research, Mumbai, India; ^2^ICMR Biomedical Informatics Centre, National Institute for Research in Reproductive Health, Indian Council of Medical Research, Mumbai, India

**Keywords:** C-tail, extracellular loops, FSH receptor, intracellular loops, receptor function

## Abstract

Follicle-stimulating hormone receptor (FSHR) is a leucine-rich repeat containing class A G-protein coupled receptor belonging to the subfamily of glycoprotein hormone receptors (GPHRs), which includes luteinizing hormone/choriogonadotropin receptor (LH/CGR) and thyroid-stimulating hormone receptor. Its cognate ligand, follicle-stimulating hormone binds to, and activates FSHR expressed on the surface of granulosa cells of the ovary, in females, and Sertoli cells of the testis, in males, to bring about folliculogenesis and spermatogenesis, respectively. FSHR contains a large extracellular domain (ECD) consisting of leucine-rich repeats at the N-terminal end and a hinge region at the C-terminus that connects the ECD to the membrane spanning transmembrane domain (TMD). The TMD consists of seven α-helices that are connected to each other by means of three extracellular loops (ELs) and three intracellular loops (ILs) and ends in a short-cytoplasmic tail. It is well established that the ECD is the primary hormone binding domain, whereas the TMD is the signal transducing domain. However, several studies on the ELs and ILs employing site directed mutagenesis, generation of chimeric receptors and *in vitro* characterization of naturally occurring mutations have proven their indispensable role in FSHR function. Their role in every phase of the life cycle of the receptor like post translational modifications, cell surface trafficking, hormone binding, activation of downstream signaling, receptor phosphorylation, hormone–receptor internalization, and recycling of hormone–receptor complex have been documented. Mutations in the loops causing dysregulation of these processes lead to pathophysiological conditions. In other GPHRs as well, the loops have been convincingly shown to contribute to various aspects of receptor function. This review article attempts to summarize the extensive contributions of FSHR loops and C-terminal tail to its function.

## Introduction

**Figure 1:**
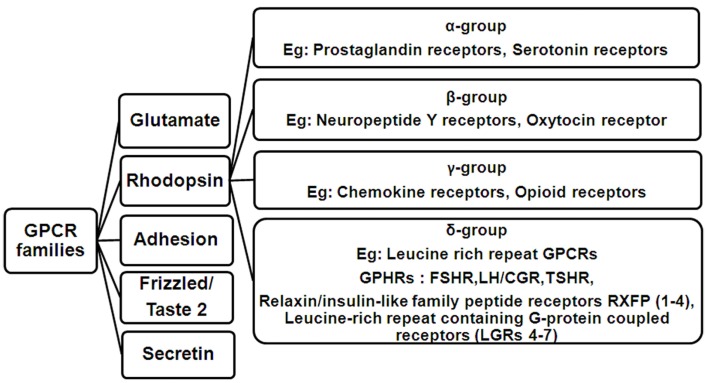
**Flowchart showing the classification of GPCR families**. The rhodopsin family consists of four main groups designated as alpha (α), beta (β), gamma (γ), and delta (δ). Members of the delta group include the Leucine-rich repeat GPCRs comprising of the three glycoprotein hormone receptors (GPHRs: FSHR, LH/CGR, and TSHR), Relaxin/insulin-like family peptide receptors RXFP (1–4) and Leucine-rich repeat containing G-protein coupled receptors (LGRs 4–7).

The ability of all organisms to receive external stimuli in the form of light, water, sound, hormones, odors, to name a few, is essential to bring about a necessary physiological response. This process is mediated through cell surface receptors, mainly the G-protein coupled receptors (GPCRs), which form the largest and most diverse class of receptors. GPCRs form a repertoire of about 800 receptors and are the largest set of drug targets in the market, thus signifying the importance of their study in greater detail. The GPCR superfamily is composed of five major families as defined by phylogenetic analysis: glutamate, rhodopsin, adhesion, frizzled/taste2, and secretin, which constitute the GRAFS classification system (1). All GPCRs are characterized by a common structure consisting of a ligand binding extracellular domain (ECD) and a signal transducing transmembrane domain (TMD) consisting of seven alpha helices spanning the membrane. The helices are connected to each other by means of three extracellular loops (ELs) and three intracellular loops (ILs) and end in a cytoplasmic tail. The GRAFS system family members differ in the sizes of their ECDs, ranging from 60 to 80 residues for growth hormone releasing hormone and calcitonin receptors in Family S, to 280–580 residues for metabotropic, glutamate receptors in Family G. Rhodopsin Family R, receptor ECD sizes vary considerably, as this is the largest family and is subdivided into four groups designated as alpha, beta, gamma, and delta (2). The delta group consists of the glycoprotein hormone receptors (GPHRs), namely, the follicle-stimulating hormone receptor (FSHR), luteinizing hormone/choriogonadotropin receptor (LH/CGR), and thyroid-stimulating hormone receptor (TSHR). A hallmark of GPHRs is the presence of a large ECD of nearly 350–400 residues containing leucine-rich repeats (LRRs), which mediate ligand binding with high affinity and specificity (3). Several other receptors also harbor the horseshoe-shaped LRR structure, which facilitates high-affinity ligand binding, e.g., LGR 4–7 (4) and some members of the relaxin family peptide receptors, namely RXFP1 and RXFP2 (5). Figure 1 is a diagrammatic representation of the GPCR families showing the delta group of Rhodopsin family containing leucine-rich repeat GPCRs.

The glycoprotein hormones include the gonadotropins, follicle-stimulating hormone (FSH), luteinizing hormone (LH), the placental hormone chorionic gonadotropin (CG), and the non-gonadotropin thyroid-­stimulating hormone (TSH). They belong to the cystine-knot growth factor superfamily and share a common heterodimeric structure composed of two non-covalently associated α and β subunits (6). The α subunits are common for all the hormones, whereas the β subunits confer functional specificity. All the glycoprotein hormones bind to their cognate GPHRs to elicit specific biological effects. FSH binds to FSHR expressed on the granulosa cells of the ovary in females, to bring about follicular maturation (7) and on the Sertoli cells of the testis in males, where it maintains the Sertoli cell population and sperm production (8).

Although a lot of research has been focused on the ECD and TMD of FSHR, emerging evidence suggests the importance of the ELs and ILs of the receptor in its function. In spite of this, a compendium of available data on the role of the loops and especially the ELs of FSHR is lacking. Hence, in this review article, we have discussed the involvement of the loops in many FSHR functions like cell surface trafficking, hormone binding, signal transduction, internalization, and recycling of the hormone–receptor complex. A few relevant examples from studies on the loops of LH/CGR and TSHR have also been cited.

## Life Cycle of FSH Receptor

**Figure 2:**
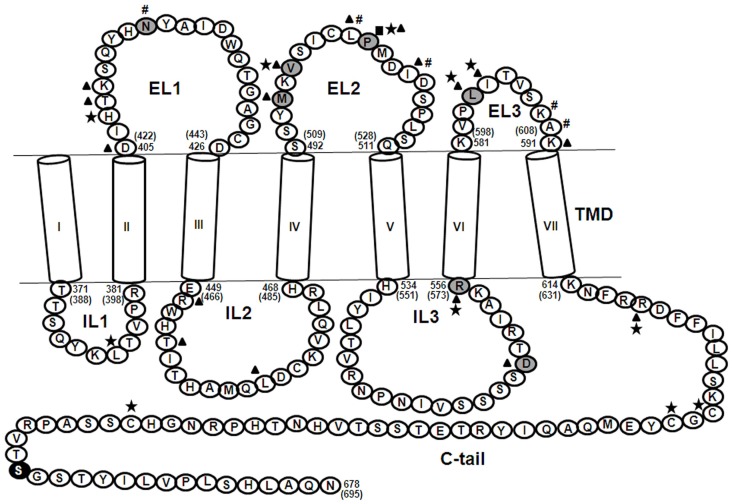
**Partial sequence of the human FSHR showing the TMD**. The seven alpha helices, shown as cylinders designated, I–VII, are connected by means of three ELs and three ILs and ends in a short C-tail. Shaded residues (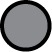) are naturally occurring mutations, (■) indicates residues crucial for cell surface receptor trafficking, ★) indicates residues important for FSH binding, (▲) indicates residues important for FSH-induced cAMP production, (#) indicates residues crucial for internalization of FSH-FSHR complex, and (●) indicates SNP. Explanation for each of these residues is provided in the text wherever applicable. The numbering system followed is according to that for the mature receptor without the 17 amino acid residue signal peptide. The numbers in parentheses correspond to the amino acid residue number including the signal peptide. The ECD comprising residues 1–349 is not shown.

The life cycle of the FSH receptor, like all other GPCRs, includes post translational modifications like glycosylation, palmitoylation, and also formation of higher order oligomers in the ER and Golgi networks, after which the mature receptor is trafficked to the cell surface (9). Abell et al. (10) have shown that deletion mutants of ELs of LH/CGR result in the mutant receptor being trapped intracellularly showing the importance of ELs in cell surface receptor trafficking. In the case of the FSHR, once the mature receptor is localized on the surface of target cells, FSH first binds to the high-affinity leucine-rich LRR domains of FSHR, which results in additional interactions at the hormone–receptor interface and formation of a sulfated Tyr pocket into which the FSHR sulfated Tyr^335^ is inserted, eventually resulting in receptor activation (11). This interaction of the ligand with the ECD and relay of the signal to the TMD is probably mediated by the ELs of the FSHR. Ji et al. (12) carried out an elegant study wherein FSHR mutants, which were either binding deficient or signaling deficient, were co-expressed. It was seen that FSHR ECD (of signaling deficient mutant which was capable of binding FSH) could transactivate non-binding FSHR mutants to bring about cAMP or IP production but not both. This hormone bound to the ECD probably contacts the ELs of the receptor to bring about its activation. In TSHR, co-operative signal amplification of constitutively activating mutations (CAMs) in ELs was shown by Kleinau et al. (13) by combining the CAMs and studying the effects *in vitro*. This proved that switching of the receptor from an inactive to an active conformation takes place by means of several contacts involving both the ECD and the ELs and this is the case for FSHR too. Binding of FSH to FSHR triggers several downstream signaling pathways other than the canonical cAMP/PKA pathway, such as the protein kinase B (PKB/Akt) and serum and glucocorticoid-induced kinase (Sgk) (14), p38 MAPK (15), ERK1/2 (MAPK3/1) (16), and IP3 production (17). The receptor is then uncoupled from the G-protein, the desensitized hormone–receptor complex becomes internalized, following which most of the complex is recycled back to the cell surface and a small fraction is routed to lysosomes for degradation (18). Reports from naturally occurring and induced mutations of residues in the ELs and ILs of FSHR provide evidence for the roles of each loop in various aspects of receptor function (Figure 2).

## Extracellular Loop 1

Alanine scanning mutagenesis of the first five amino acids in extracellular loop 1 (EL1) of FSHR showed that a His^407^Ala mutation decreased FSH binding affinity, whereas substitutions at Asp^405^, Thr^408^, and Lys^409^ abolished cAMP production (19). The revertant mutants showed FSH binding and cAMP production similar to WT indicating the importance of EL1 residues in FSH binding and signaling. Casas-González et al. (20) reported a novel activating FSHR mutation N^431^I in EL1 in a male who exhibited normal spermatogenesis but low-serum FSH levels. The mutation impaired the desensitization and internalization of the hormone–receptor complex due to its inability to recruit beta arrestin proteins, which mediate internalization, as well as affected the recycling of the complex, as studied by pulse chase assays. In case of TSHR, several activating mutations have been identified. One such mutation in the ECD that has been studied in detail is S^281^T/I/N (21). Ala substitution mutagenesis studies on the aromatic residues in the vicinity of S^281^, proximal to TSHR EL1, revealed that mutation of Y^481^in EL1 along with the surrounding aromatic residues was shown to affect receptor signaling. Antipeptide antibodies corresponding to EL1 region (residues 405–426) of FSHR could detect the receptor as determined by flow cytometry as well as inhibit FSH binding and cAMP production in a dose-dependant manner (22). Thus, FSHR EL1 residues are probable secondary hormone binding sites and important in FSH binding, cAMP signaling, internalization, and recycling of hormone–receptor complex.

## Extracellular Loop 2

**Figure 3:**
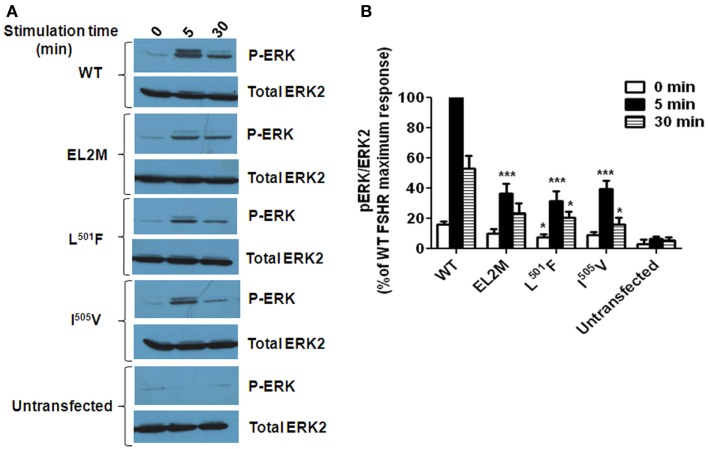
**FSH-induced ERK phosphorylation in wild type (WT) FSHR, chimeric EL2M FSHR, and the point mutants L^501^F and I^505^V (31)**. **(A)** Representative western blot showing the levels of phospho ERK (P-ERK) and total ERK2 in cell lysates from HEK293 cells transiently expressing the FSHR constructs and stimulated without or with 100 ng FSH for 5 or 30 min. Untransfected cells served as a negative control. **(B)** Densitometric analysis showing the ratio of phosphorylated ERK: total ERK2. The maximum response obtained by WT FSHR at 5 min post stimulation with 100 ng FSH was considered to be 100% and the % response obtained for the mutants at 0, 5, and 30 min post FSH induction (100 ng) was determined by comparing it with the maximum response and plotted. The value of *P < 0.05 and ***P < 0.001 with respect to WT FSHR was considered to be statistically significant.

**Figure 4:**
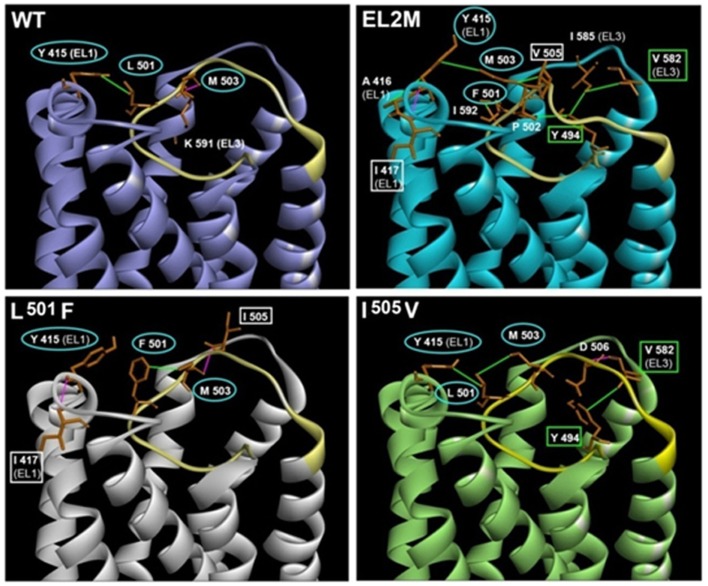
**Structural models of the TMD and ELs of WT FSHR, chimeric EL2M FSHR, and the point mutants L^501^F and I^505^V (31)**. The acquired interactions in chimeric EL2M FSHR and point mutants L^501^F and I^505^V with the adjacent residues in EL1 and EL3, which are absent in WT FSHR is depicted. EL2 region in all the models is shown in yellow. The residues involved in interaction in all the four models are circled in cyan (Y^415^, L^501^/F^501^, and M^503^). Residues showing similar interaction in chimeric EL2M and L^501^F mutant are shown in white square boxes (I^417^ and I^505^/V^505^). Residues showing similar interaction in chimeric EL2M and I^505^V are shown in green square boxes (Y^494^ and V^582^). Hydrogen bonds and hydrophobic interactions are depicted in pink and green lines. The models were built using Discovery Studio 3.5 and minimized using Schrodinger 2013 OPLS 2005 force field with default parameters, as described in Banerjee et al. (31).

As in most other GPCRs, extracellular loop 2 (EL2) plays an indispensable role in FSHR function. Chimeric receptors of FSHR ECD/TMD and C-tail of the *Drosophila melanogaster* fly receptor LGR2 had high-basal cAMP levels suggesting constitutive activation of receptor due to removal of the constraint imposed by the interaction of exoloops with the ECD (23). In the case of the LHR, this constraint was imposed by EL2, and hence, it is possibly true for EL2 of the FSHR too. Meduri et al. (24) reported a novel homozygous mutation Pro^519^Thr in a patient with primary amenorrhea. The mutation at this highly conserved Proline residue resulted in the inability of the mutant receptor to traffick to the cell surface and subsequently abolished FSH binding and cAMP production. Since the receptor was trapped intracellularly, follicular maturation was blocked, resulting in the clinical manifestation of premature ovarian failure. Functional characterization of a novel heterozygous mutation M^512^I in a woman with spontaneous ovarian hyperstimulation syndrome (sOHSS): ovarian enlargement due to several luteinized cysts within the ovaries due to abnormally high levels of hCG in pregnancy (25) or sometimes due to high levels of TSH (26) revealed that the mutation impaired cAMP signaling and PI3K/AKT pathways (27). Recently, a novel mutation Val^514^Ala was identified in a patient undergoing IVF who exhibited symptoms of iatrogenic ovarian hyperstimulation syndrome (aOHSS): excessive follicular recruitment and enlargement due to ovarian stimulation with exogenous FSH during ART (28). The mutation at this conserved Val residue conferred higher cell surface receptor expression, higher FSH binding, and attained saturation of cAMP production at low doses of FSH as compared to wild type receptor (29). Both the Pro^519^ and Val^514^ residues, mentioned here, are not only conserved across FSHR of all species but also across LHR and TSHR, indicating their importance. The significance of FSHR specific, that is, non-conserved residues of EL2 of FSHR was demonstrated by swapping six FSHR specific residues in EL2 with those from LH/CGR (30). The chimeric EL2M receptor had an impaired cAMP response as well as reduced internalization of the FSH–FSHR complex. Further, characterization of six individual substitution mutants of the FSHR specific residues of EL2 was performed and it was found that a L^501^F mutant showed weak interaction with beta arrestins consistent with its low internalization, impaired FSH-induced cAMP response, as well as low levels of ERK phosphorylation (31). The I^505^V substitution also affected receptor function to some extent. Figure 3 shows the low levels of ERK phosphorylation in chimeric EL2M and the point mutants L^501^F and I^505^V as compared to WT FSHR as reported in Banerjee et al. (31). Molecular modeling studies revealed that the L^501^F and I^505^V substitutions in EL2 resulted in gain of interactions in the mutant receptors as compared to wild type receptor (Figure 4). Mutations in EL2 of LHR have also been reported to either enhance internalization and cAMP signaling (F^515^A and T^521^A) or impair internalization (S^512^A and V^519^A) and cAMP signaling (32) indicating the importance of ELs of GPHRs in agonist-induced internalization of the hormone–receptor complex. Thus, FSHR EL2 residues are essential for cell surface receptor trafficking, FSH binding, cAMP/ERK pathway/PI3K pathway, internalization of FSH–FSHR complex, and beta arrestin recruitment.

## Extracellular Loop 3

Extracellular loop 3 (EL3) is the shortest EL of FSHR consisting of only 11 amino acid residues compared to 22 for EL1 and 20 for EL2. *In vitro* characterization of a mutated FSH receptor in a compound heterozygous patient (Asp^224^Val in ECD and Leu^601^Val in EL3), with POF was carried out by Touraine et al. (33). Although the cell surface FSHR expression and FSH binding affinity of Leu^601^Val mutant were similar to wild type receptor, this substitution impaired cAMP response, which might have led to a block in follicular maturation beyond the early antral stage. Contrary to this observation, Ryu et al. (34) and Sohn et al. (35) showed that substitution/deletion of Leu^583^ [same as Leu^601^ reported by Touraine et al. (33)] and Ile^584^with a panel of amino acids enhanced FSH binding. However, substitutions at Leu^583^, Ile^584^, and Lys^590^ abolished cAMP, consistent with the study carried out by Touraine et al. (33), thus clearly indicating the importance of EL3 residues in cAMP signal transduction. Sohn et al. (35) also showed that substitution at Leu^583^with the aromatic amino acids Phe or Tyr, improved the hormone binding and cAMP induction, but impaired inositol phosphate (IP) production. IP induction was also found to be abolished for Ile^584^ and Lys^590^ substitutions. Photoaffinity labeling studies revealed that interaction of FSH–FSHR takes place through contact of FSH beta with the N-terminal ECD, whereas the FSH alpha subunit is oriented toward EL3, indicating the important role of this loop in hormone–receptor interaction (36). Along with EL1, antipeptide antibodies corresponding to EL3 (FSHR residues 581–591) were also found to be surface accessible and capable of inhibiting hormone–receptor interaction as determined by radioreceptor assay and cAMP assay (22). This information along with the data obtained by the study conducted by Sohn et al. (36) indicate that the ELs of FSHR probably serve as secondary hormone binding sites by means of their interaction with the alpha subunit of FSH. In the TSHR, Claus et al. (37) showed that a hydrophobic cluster in the center of EL3 is essential for cAMP signaling as seen by the loss of signal generation after mutation of residues 652–656, comprising the cluster. Another interesting observation with respect to the role of EL3 in receptor function was obtained by generation of FSHR/LH-CGR chimeric receptors, as mentioned earlier (30). Substitution of the three FSHR specific residues in EL3 of FSHR with the corresponding residues in LH/CGR resulted in the chimeric EL3M receptor exhibiting higher internalization of FSH–FSHR complex without any change in the affinity for hormone binding. The cAMP signaling response, however, was comparable to that of wild type receptor. Pulse chase experiments revealed that recycling of the chimeric EL3M receptor was affected. Thus, FSHR EL3 residues seem to be essential for FSH–FSHR interaction, FSH binding, FSH-induced cAMP and IP production and internalization, and recycling of FSH–FSHR complex.

## Intracellular Loop 1

Phosphorylation of FSH receptor post ligand stimulation was reported by Quintana et al. (17). Phosphorylation is mediated by G-protein-related kinases (GRKs), mainly GRK2 and GRK6, in the case of FSHR (38). The phosphorylated receptor then recruits adaptor proteins called beta arrestins, which help in mediating the internalization of the hormone–receptor complexes via clathrin coated pits (39). Generation of a mutant rFSHR-1L where the S/T residues in intracellular loop 1 (IL1) of rat FSHR were mutated (T^369^I, S^371^I, T^376^N of IL1) greatly affected the phosphorylation of the receptor even though FSH binding affinity was unaffected and basal cAMP response of mutant receptor was higher (constitutively active) (40). This study showed that phosphorylation of IL1 of FSHR is required both for uncoupling of receptor in response to FSH stimulation and its internalization. Further, abolishing the phosphorylation sites at IL1 was shown to affect beta arrestin-2 recruitment, hence receptor internalization (41). Nechamen et al. (42) have also shown interaction of the APPL1 (adaptor protein containing PH domain, PTB domain, and leucine zipper motif) with hFSHR IL1 and that it links the FSH-stimulated receptor to the PI3K/Akt pathway essential for survival of the dominant follicle. Alanine scanning mutagenesis of IL-1 residues demonstrated that L^377^A and F^382^A mutants showed low-FSH binding, whereas K^376^A showed FSH binding and cAMP production similar to wild type FSHR (43). However, the K^376^A mutation in FSHR inhibited its interaction with the adaptor protein APPL1 and abrogation of this interaction blocked FSHR-mediated inositol 1, 4, 5-trisphosphate (IP3) induction and FSH-induced calcium signaling. Thus, FSHR IL1 residues seem to be crucial for FSH binding, FSH-induced PI3K pathway, interaction with APPL1 protein to bring about IP3 production, receptor phosphorylation, and interaction with beta arrestins to mediate internalization.

## Intracellular Loop 2

A yeast-based interaction trap assay identified the interaction of intracellular loop 2 (IL2) of FSHR with 14-3-3 tau protein, which is important for ER localization of membrane proteins (44). Scanning alanine mutagenesis of the IL2 residues ^447^TLE^449^ and ^450^RWH^452^ resulted in loss of this interaction with 14-3-3 tau protein thus identifying the residues crucial for this interaction (45). Despite normal FSH binding, the H^452^A mutant showed low levels of internalization and no cAMP production indicating the importance of IL2 residues. IL2 of FSHR bears the highly conserved class A GPCR ERW motif, which is crucial for receptor activation (46). Timossi et al. (47) generated minigene constructs of three ILs of human FSHR and found the IL2 to be essential for Gs coupling and cAMP production. Minigene encoding free IL-2 as well as minigene IL2 mutants R^467^A and R^467^K and co-expression of full length WT with minigene mutant L^477^A lowered FSH stimulated but not basal cAMP levels. Further full length IL2 mutants were made and it was found that FSH binding to these mutants was moderately affected in the constitutively active mutants L^477^A/D/P and to a lesser extent in L^477^K/R mutants. Full length FSHR IL2 mutants R^467^A/H, T^470^A abolished FSH-induced cAMP production without altering basal levels, L^477^A/D/P mutations led to elevated basal cAMP levels, L^477^K/R mutants showed less FSH-induced cAMP production. The FSHR-2L mutant, in which the two threonine of IL2 were subjected to alanine substitution (Thr^451^Ala, Thr^453^Val), and a rFSHR-(2L + 3L) mutant, in which the two threonines in the second IL along with the seven Ser/Thr residues in the intracellular loop 3 (IL3) were substituted with alanine residues (Thr^451^Ala, Thr^453^Val, Thr^536^Ala, Thr^541^Ala, Ser^544^Ala, Ser^545^Ala, Ser^546^Ala, Ser^547^Ala, Thr^549^Ala), were deficient in phosphorylation, bound FSH with comparable affinity to WT but showed low levels of FSH-induced cAMP production (inactivating mutations) (48). Further, in this study, it was shown that rFSHR-D^389^N and rFSHR-Y^530^F (D and Y are highly conserved residues across GPCRs), two inactivating mutations possessing intact phosphorylation sites showed impairment in phosphorylation. Overexpression with GRK-2 was shown to rescue phosphorylation of both the mutants but internalization of only D^389^N mutant, whereas overexpression of arrestin-3 (β-arrestin 2) could rescue internalization of both mutants providing the first evidence of the role of beta arrestins in FSH-mediated receptor internalization post receptor phosphorylation with GRKs. In LHR too, mutagenesis studies revealed several residues in IL2, like Lys^455^ and His^460^, to be essential for ligand binding and Glu^441^ and His^460^ to be important for cAMP response (49). Thus, FSHR IL2 residues are especially important for cAMP production and also for receptor phosphorylation.

## Intracellular Loop 3

A synthetic peptide corresponding to residues 533–555 of IL3 of the rat FSHR was shown to inhibit cAMP production and estradiol synthesis in cultured Sertoli cells from immature rat testes (50). Along with IL1, mutations in IL3 residues also were shown to affect receptor phosphorylation; however, the effect was not as pronounced as in IL1 mutation. The rFSHR-3L mutant, in which the Ser/Thr residues in IL3 were mutated to alanine (Thr^536^Ala, Thr^541^Ala, Ser^544^Ala, Ser^545^Ala, Ser^546^Ala, Ser^547^Ala, Thr^549^Ala of IL3)and a rFSHR-(3L + CT) mutant, in which the Ser/Thr residues in both IL3 and C-tail were mutated to alanine (Thr^536^Ala, Thr^541^Ala, Ser^544^Ala, Ser^545^Ala, Ser^546^Ala, Ser^547^Ala, Thr^549^Ala, Ser^624^Ala), showed unaltered FSH binding, whereas rFSHR-3L was found to be a constitutively active mutant, as it exhibited high-basal cAMP response (40). The mutant receptors affected the phosphorylation of FSHR to some extent, as well as its uncoupling from adenylyl cyclase enzyme without affecting its internalization. Using chimeric FSHR and LHR receptors, the interaction of threonine residues in IL3 with beta arrestin proteins, and hence, internalization of these gonadotropin receptors was shown by Bhaskaran et al. (51). GPCRs harbor a BBXXB motif (where B represents a basic amino acid whereas any amino acid can be presented at “X”) in the intracellular domains, which is an essential determinant of receptor activation. FSHR contains a reverse BXXBB situated at the juxtamembrane region of IL3and C tail, which was subjected to Ala substitution mutagenesis (52). All the IL3 mutant receptors BXXAB, AXXBB, and BXXBA showed FSH binding similar to WT, but binding was affected in the same three mutants of C-tail. However, cAMP production was abolished in AXXBB and BXXBA mutants but was normal in BXXAB mutant of IL3. In the case of the C-tail, cAMP production of all three mutants was affected. Thus, it appears that the BXXBB motif at the IL3 of the hFSHR is essential for Gαs coupling and cAMP production, whereas the same motif in the C-tail is more important for membrane expression as the mutation resulted in an immature form of the receptor, which was unable to bind the hormone. An interesting study by Cohen et al. (53) identified IL3 to be a site of FSHR ubiquitination by a yeast two-hybrid screen. However, mutating the only Lys residue available for ubiquitination (K^555^R) did not disrupt FSHR–ubiquitin interaction indicating that other determinations of receptor ubiquitination exist. The importance of this loop is further emphasized by the presence of naturally occurring mutations in the loop that affect receptor function. Beau et al. (54) reported that a woman with secondary amenorrhea and high-serum FSH levels was found to harbor two FSHR mutations: Ile^160^Thr (ECD) and Arg^573^Cys (IL3). The mutation in the ECD affected cell surface receptor expression, whereas the IL3 mutation impaired cAMP signaling. A constitutively active mutation D^567^G was identified by Gromoll et al. (55) in a hypophysectomized man. Later in 2003, Smits et al. (56) reported a mutation at the same position in FSHR IL3 in a woman with spontaneous OHSS. The D^567^N substitution in this case conferred high-basal cAMP response as well as loss of functional specificity as the mutant receptor showed a dose-dependent increase in cAMP production upon hCG or TSH stimulation. The importance of this residue is further corroborated by the fact that it lies in a protein kinase CK2 consensus site and brings about phosphorylation of adaptor proteins like beta arrestins, which mediate receptor internalization (57) via their interaction with FSHR (58). Kluetzman et al. (59) showed that both the naturally occurring D^550^G (same as D^567^G) mutation and the alanine substituted mutation D^550^A showed accumulation of FSH in mutant receptors in intracellular stores due to decreased degradation after internalization as evidenced by radioreceptor assay as well as visualization by confocal microscopy. Thus, FSHR IL3 residues seem to be important for receptor phosphorylation, cAMP response as well as ubiquitination.

## C-Terminal Tail

As in all other GPCRs, the carboxy-terminal tail bears the highly conserved F(X)_6_LLmotif (where X can be any residue, and L is leucine or isoleucine), which is important for trafficking of the mature receptor from ER to the cell surface (60). This motif is located between 616 and 624 residues in the mature FSH receptor (9). Another important post translational modification in GPHRs is palmitoylation of the cysteine residues and this is crucial for receptor endocytosis and other post endocytic events (61). Two conserved cysteine residues (at positions 629 and 655) and one non-conserved Cys residue (at position 627) are present in the C-tail of human FSHR, which are potential sites for S-acylation with palmitic acid, were investigated by Uribe et al. (62). Low-FSH binding in C^629^A, double mutants C^627^/^629^A and C^629^/^655^A, and the triple C^627^/^629^/^655^A receptor mutants without change in FSH binding affinity was observed due to low-cell surface FSHR expression of mutants, whereas low internalization for FSH–FSHR complex of Cys^655^A/S/T mutants was seen. C^629^A, C^655^A/S/T, the double mutant C^627^/^629^A, and the triple mutant C^627^/^629^/^655^A showed low-cAMP production. The triple mutant C^627^/^629^/^655^A did not show palmitoylation indicating the importance of all the three residues for palmitoylation. Single-alanine substitutions of residues in the IL3 of TSHR also revealed that the residues were crucial for G-protein activation (63). Truncation mutants of the C-terminal tail (removal of the last eight residues) of hFSHR and rFSHR decreased the amount of internalized ^125^I-hFSH (18). Confocal microscopy analysis showed that in contrast to the internalized WT receptors, which localized only to endosomes, the internalized truncated receptors localized to both endosomes and lysosomes. This study showed that most of the FSH–FHSR complex gets recycled back to the cell surface and truncation of eight residues from the C-tail reroute a substantial portion of the internalized FSH–FSHR complex to a degradation pathway. Ala substitution of the Ser/Thr cluster T^638^A, T^640^A, S^641^A, S^642^A, T^644^A in the C-tail of rFSHR showed similar FSH binding affinity to mutant receptor as compared to WT but impaired internalization of FSH–FSHR complex (64). Loss of the Ser/Thr cluster in the mutant FSHR resulted in enhanced cAMP production due to its inability to get desensitized, impaired phosphorylation, β-arrestin recruitment, and hence, impaired internalization of FSH–FSHR substantiated the importance of the C-tail in FSHR function. Thomas et al. (65) reported that FSHR forms oligomers in a constitutive manner before coming to the cell surface and discovered during the course of this study that the C-terminal epitope tags undergo proteolytic processing, so such C-terminal tagged receptors could not be exploited to study receptor oligomerization. To overcome this shortcoming, Mazurkiewicz et al. (66) generated chimeras of FSHR and extreme C-tail fluorescent fusion proteins: FSHR-LHRcT-YFP/FSHR-LHRcT-mCherry pairs possessing amino acid residues 1–611 of the hFSHR and residues 604–674 of the rLHR. Fluorescence correlation spectroscopy and photon counting histogram studies with these chimeric FRET pairs demonstrated the presence of freely diffusing FSHR homodimers on the surface of live cells. FRET experiments also demonstrated that the hFSHR-rLHR-cT chimera formed hetero-dimers/hetero-oligomers with LHR and this possibly occurred during granulosa cell differentiation. Zariñán et al. (67) had reported that co-transfection of WT FSHR with mutants R^556^A (IL3) or R^618^A (C-tail) showed dose-dependent inhibition in FSH binding and cAMP production with increasing amounts of mutant DNA and subsequently rescue of function by co-transfection with WT fragments of TMD 5, 6, or 7 and/or C-tail suggesting oligomerization of FSHR. The crucial role played by the C-tail in receptor function is reinforced by the existence of a SNP p.Asn^680^Ser (rs6166), which has been studied extensively in various ethnic groups across the world and is believed to serve as a marker to predict ovarian response in women undergoing assisted reproductive technology programs (68–74). Thus, the C-tail of FSHR plays an indispensable role in palmitoylation, cell surface receptor trafficking, receptor phosphorylation, interaction with beta arrestin proteins, and hence, internalization of FSH–FSHR complex.

## Conclusion and Future Directions

Genetic alterations in GPCRs, resulting in loss or gain of function, lead to several pathological conditions and are being studied by several groups to develop drugs targeted at the receptor to rescue its function (75, 76). Knowledge of combination of SNPs in FSH beta and FSHR is essential for determining patient risk/treatment outcome and for designing treatment in cases of infertility (77). Also, *in vitro* studies on several naturally occurring mutations [reviewed by Desai et al. (78)] and studies on both FSH beta and FSHR knockout mice (79) indicate the pivotal role of this ­hormone–receptor interaction, failure of which leads to reproductive dysfunctions. Owing to the large size of the receptor, its interaction with FSH takes place at several discrete regions on the ECD and this hormone–receptor ECD complex possibly makes contacts with the ELs and the signal is then relayed downstream through the TMD. Other than mutations in the ECD and TMD, which may result in intracellular retention of the receptor, its inability to bind FSH or bring about signal transduction, several such mutations in the loops display similar characteristics and this necessitates their study in greater detail. Therefore, in order to develop drugs targeted to rescue the function of the receptor, a thorough understanding of the epitopes crucial for FSH–FSHR interaction is a must. In recent times, several small molecule FSHR agonists, which can be orally administered like Org 214444-0 (80), FSHR allosteric modulators like Compound 5 (81) have been developed, which can be administered to patients for ovulation induction. Pharmacoperones (pharmacological chaperones that assist in folding and routing of mutant receptors to the cell surface) like Org 41841 have been shown to rescue the function of the A^189^V FSHR mutant, which was trapped intracellularly and hence exhibited low-cell surface FSHR expression (82). As these molecules hold great therapeutic potential, the understanding of the biochemical mechanism of their interaction with the receptor and identification of sites of interaction with the receptor is imperative. Along with the ECD and TMD, detailed analysis of the residues in the ELs and ILs is therefore of utmost importance given their versatile roles in FSH–FSHR interaction.

## Conflict of Interest Statement

The authors declare that the research was conducted in the absence of any commercial or financial relationships that could be construed as a potential conflict of interest.
